# Study on the Psychological Effects of Intangible Cultural Heritage Advertising with Different Degrees of Situational Involvement

**DOI:** 10.3390/bs14070623

**Published:** 2024-07-22

**Authors:** Ruiying Kuang, Changping Hu, Shiyu Huo, Yitian Shi, Xinai Tang, Lulu Mao

**Affiliations:** 1 Department of Visual Communication Design, Changsha Insitute of Technology, Changsha 410000, China; kuangruiying@xtu.edu.cn; 2 College of Humanities and Arts, Hunan University of Finance and Economics, Changsha 410000, China; 3 Art Insitute, Xiangtan University, Xiangtan 411100, China; hcp0708@xtu.edu.cn (C.H.); 202321072059@smail.xtu.edu.cn (S.H.); 202005430504@smail.xtu.edu.cn (Y.S.); 202005430518@smail.xtu.edu.cn (X.T.)

**Keywords:** intangible cultural heritage advertisement, psychological effects, situational involvement, emotional advertisement, rational advertisement

## Abstract

This review addresses the issues of low consumer engagement and market development difficulties in intangible cultural heritage (ICH) products. Dietary ICH products are selected as research materials to discover contemporary commercial survival paths for ICH through the psychological effects of advertising. Firstly, this study examines the respective advantages of rational and emotional ICH advertisement in terms of emotional responses, cognitive responses, attitudes, recall, and recognition. Then, it explores the effects of different ICH advertisement types (rational advertisement, emotional advertisement) and different degrees of situational involvement (purchasing for oneself, purchasing gifts for others) on the advertising effectiveness, aiming to identify factors influencing the psychological effects of ICH advertisement. Through statistical analysis, the main conclusions are as follows: (1) Rational ICH advertisement prompts consumers to consider the actual attributes of ICH products, leading to a more positive purchasing attitude. (2) Emotional ICH advertisement is more effective in eliciting positive emotions from consumers and enhancing brand memory. (3) Under the scenario of purchasing a gift for others, emotional ICH advertisement has a more positive impact on consumers’ attitudes towards advertising. (4) Under different degrees of situational involvement, rational ICH advertisement has a more positive impact on consumers’ purchasing attitudes. This study not only provides guidance for optimizing ICH advertising strategies but also offers new directions for market expansion, contributing valuable insights into cultural heritage preservation, as well as the development and protection of ICH.

## 1. Introduction

According to the UNESCO Convention for the Safeguarding of the Intangible Cultural Heritage, intangible cultural heritage (ICH) refers to the various social practices, representations, expressions, knowledge, and skills, as well as the instruments, objects, artifacts, and cultural spaces associated therewith, that communities, groups, and, in some cases, individuals recognize as part of their cultural heritage [1]. Although ICH carries historical and cultural information, as well as the traditional craftsmanship of human beings, it gradually loses its local market under the powerful effects of time and encounters obstacles everywhere as globalization progresses. As the economist Herbert Simon put it, “With the advent of the information age, it is no longer information but attention that is valuable”. As a model of the application of the attention economy in the market economy, advertising has opened up a steady economic resource for ICH. Culturally significant ICH products that capture consumers’ attention through advertising become the “lifeblood” of ICH inheritance and development, thus forming a virtuous interactive cycle of development and protection. Therefore, it is necessary to seek new protection methods and strategies through ICH advertisement (hereinafter referred to as “ICH ad”).

ICH ad is a form of advertising that aim to preserve and promote ICH products by conveying information about their history, culture, craftsmanship, value, and characteristics through various media and communication methods. This type of advertising aims to raise public awareness and recognition of ICH; promote its protection, inheritance, and development; and support the sales of related products or services. ICH ad can be divided into rational and emotional advertisement based on the different focuses of the advertising appeals [2]. Rational advertisement (hereinafter referred to as “rational ad”) tends to highlight the practical characteristics of ICH, using straightforward copying and imagery techniques to showcase ICH. In contrast, emotional advertisement (hereinafter referred to as “emotional ad”) aims to convey messages by eliciting emotional resonance and reactions from the audience. Unlike rational ad, emotional ad does not primarily rely on rational factors such as the functionality, characteristics, or the prices of products. Instead, they attract attention and leave a lasting impression by telling touching stories and using high-quality audiovisual effects to move the audience emotionally.

In terms of China’s ICH protection and rural revitalization policies, ICH ad has led to remarkable progress in content innovation, media marketing, professional operation, and other aspects of ICH ad, resulting in an increasing number of ICH stakeholders paying attention to the importance of advertising.

### 1.1. Brand Building

The analysis of ICH brand building is primarily based on the macro policy environment and economic environment. Some scholars have proposed methods for achieving a win–win situation between ICH and a business [3,4,5]. Falguni conducted a comparative evaluation of local tourism activities in India and pointed out that advertising has become a powerful marketing tool with which to build ICH brands [6]. Zuo analyzed the promotional subjects and strategies of existing ICH brands and proposed brand diversification, as well as innovation strategies [7]. Hui analyzed the current status of the innovative development of Chinese ICH brands and proposed strategies to address the over-commercialization of ICH brands [8]. These scholars believe that ICH ad can enhance the cultural value and influence of brands while having a profound impact on consumers’ emotions and perceptions. Moreover, the government, enterprises, and individual practitioners are involved in ICH brand building. For example, the ICH ad project of Hunan Satellite TV in China relies on media influence to build ICH brands into business cards and promote the sound ecological development of the industry [9]. Portugal incorporates ICH into cities’ branding strategies and policy internationalization to enhance their images [10].

### 1.2. Advertising Performance

The evaluation of advertising performance must take the characteristics and values of ICH and ways to effectively integrate these elements into advertising into account. Zhang took the public service advertisement of the 24 Solar Terms on CCTV as an example and believed that ICH narration is conducive to building national cultural confidence [11]. Zhao, starting from the three communication subjects of inheritors, commodity brands, and public welfare themes, sorted and analyzed the expression rules and preferences of the art used in different ICH [12]. Li integrated Cantonese opera costume elements into ICH ad to promote the inheritance and innovation of cultural heritage [13]. Qi systematically analyzed different dimensions of ICH tourism advertising experiences and their relationships with participants’ emotional reactions, providing new theoretical and practical perspectives for heritage tourism research [14]. To further explore advertising effectiveness, Salucci tested video and photo advertisements through the methods of approach, progression, and experience to provide more possibilities for other academic evaluations [15,16]. Jiang found that incorporating interactive narrative designs into advertising can better promote the dynamic inheritance and development of Jiangnan Sizhu, an ICH project [17].

### 1.3. Propagation Model

Research has focused on exploring the innovation of ICH ad’ communication modes in the digital and intelligent creative economy and seizing the opportunity for modernization [18,19]. Eugene paid attention to the symbiotic relationship between ICH and its audience and used a modern media language to design and transform IP derivatives from ICH resources [20,21]. Ximeng believed that films and TV dramas energize ICH elements and consolidate their cross-cultural dissemination foundation [22,23]. Guo, based on object consistency, aimed to clarify the impact of image consistency and brands on tourists’ willingness to purchase intangible cultural heritage souvenirs [24]. Xue used the analytic hierarchy process to construct a theoretical evaluation model of the digital communication effects of intangible cultural heritage [25]. Additionally, social network platforms such as TikTok and YouTube grant ICH inheritors the ability to expand their reach and spread cultural ideas to thousands of households through videos from their daily life, crafts, and performance videos [26,27,28,29].

However, the research on ICH ad is still in the initial stage and requires further in-depth discussion. Whilst we carried out our field investigation, several inheritors described their predicaments. The first dilemma is a lack of product innovation: in recent years, major universities and design companies have begun to create innovative ICH products, but most have ended up as failures, and once the designer has left, the inheritors still sell the original traditional products. This is mainly due to the mismatch between high development and production costs and a low sales income, while traditional products are well known and more popular with tourists. The second dilemma is a lack of effective publicity: a significant amount of money is spent without seeing any timely returns; therefore, it can be seen that there are still some problems in the existing research. On the one hand, the research on advertising performance effects based on audience needs is insufficient. Many ICH ad is created according to the advertisers’ own preferences and needs; for example, no rigorous preliminary research is carried out to understand an audience’s needs or assess their understanding and emotions, so it is difficult to ensure advertising performance. On the other hand, there are few studies on the psychological effects of ICH ad. Data proving whether an advertising message has effectively affected an audience’s psychology, attitude, and behavior and whether the advertising goal has been achieved remain scarce. 

## 2. Study 1: Comparative Study on the Advantageous Effects of Emotional and Rational ICH Ad

### 2.1. Research Aim 

In the advertising and marketing campaigns of ICH products, advertisements that can evoke emotions are increasingly favored by ICH marketers. As an important means of cultural promotion and brand image building, such emotional ad frequently appears in various media. In theoretical research, these are also increasingly valued by ICH researchers. Based on previous research results, this study compares the advantages of rational ICH ad and emotional ICH ad, exploring the differences between the two types of advertisement in influencing emotions, cognition, attitudes, and memory. The five measurement indicators for the psychological effects of advertising are cognitive thinking, emotional response, advertising attitude, purchasing attitude, and brand recognition. This provides a basis for further research.

### 2.2. Research Hypothesis

Based on domestic and international research results, emotional ad with positive emotional appeals can trigger stronger positive emotions in individuals compared with rational ad [30,31,32,33]. Emotional ad uses storytelling to evoke individual memories, which, through emotional resonance and nostalgic emotions, elicit pleasant memories related to the advertising content, thereby enhancing positive emotions towards the advertisement. It is worth noting that most audiences tend to recall happy experiences and avoid unpleasant memories during this process; thus, negative emotions are insignificant under both types of advertising. ICH advertisements primarily aim to achieve two goals: (1) to spread ICH culture and (2) to promote ICH craftsmanship. Emotional ICH ad tells the history and culture of ICH, while rational ICH ad explains the production techniques of ICH products. Therefore, we believe that this pattern is also evident in ICH ad. Based on the above analysis, this study hypothesizes the following:

**H1a.**  *Emotional ICH ad has a significantly more positive impact on participants’ emotions compared with rational ICH ad.*

**H1b.**  *Neither type of ICH ad has a significant negative effect on participants’ emotions.*

Based on previous research, the emotional experiences triggered by advertisements are transferred to the advertisements themselves, meaning that the emotional responses elicited by the advertisements directly affect the audience’s attitude towards the advertisements, which further influences their attitude towards the brand [34]. Therefore, emotional ICH ad is likely to stimulate more positive attitudes in the audience, leading to a more favorable perception and evaluation of ICH products, thereby resulting in a stronger purchasing intention for ICH products. Based on the above analysis, this study hypothesizes the following:

**H2a.**  *Emotional ICH ad has a significantly more positive effect on participants’ attitudes towards an advertisement compared with rational ICH ad.*

**H2b.**  *Emotional ICH ad has a significantly more positive effect on participants’ purchasing attitudes compared with rational ICH ad.*

Previous studies have found a significant correlation between emotional response and consumers’ attention, engagement, and memory [35]. The emotions and cognitive experiences elicited by advertisements may be reflected in the processing of advertisement content and product information. Therefore, emotional ICH ad is likely to evoke more emotional resonance from the audience, making them think more about the memories related to the advertising plot. In contrast, rational ICH ad may prompt the audience to think more deeply about the product information. Similarly, information filled with emotional experiences is prioritized in processing; thus, emotional ICH ad can improve the audience’s information processing and memory efficiency. Based on the above analysis, this study hypothesizes the following:

**H3a.**  *Emotional ICH ad has a significantly larger impact on participants’ thinking about the emotional narrative of the advertisement compared with rational ICH ad.*

**H3b.**  *Rational ICH ad has a significantly larger impact on participants’ deep thinking about the product information compared with emotional ICH ad.*

**H4.**  *Emotional ICH ad has a significantly larger impact on participants’ brand recognition accuracy compared with rational ICH ad.*

### 2.3. Research Methods

#### 2.3.1. Pretest: Control Test of Stimulus Material Selection and Advertisement Type

The research material for this study is an advertisement for Changle Sweet Rice Wine, from the Chenji brand. Changle Sweet Rice Wine is an ICH protection project in the culinary category of Yueyang City, Hunan Province. For thousands of years, the handmade craftsmanship and secret brewing techniques for this product have been passed down through generations, making it a cherished hospitality item for residents in northeastern Hunan during the Spring Festival. The reasons for selecting the Chenji brand are as follows: (1) This brand needs advertising to promote cultural heritage and traditional craftsmanship. (2) The brand has been dedicated to differentiating itself from other sweet rice wine brands, launching various products such as “easy-pack”, “gift-pack”, and “postpartum wine” to attempt to find economic value for ICH in the commercial market. (3) The brand has low brand awareness and is only sold within the county. By using a relatively unknown ICH product, this study effectively isolates the impact of emotional and rational ad on consumer attitudes and behaviors, free from the influence of pre-existing brand perceptions. In summary, the selection of Chenji Changle Sweet Rice Wine highlights the challenges faced by ICH products in modern advertising. This allows this study to explore how contemporary advertising strategies can effectively promote traditional products, providing valuable insights for the development of ICH advertisement campaigns.

**Figure 1:**
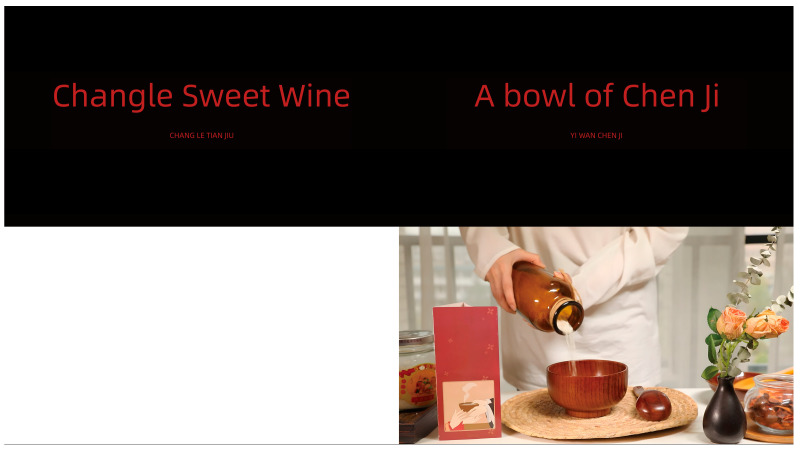
Ad 1: rational advertisement entitled “Cinnamon wine with fragrance”.

Video advertising was used as described below:

Ad 1: A rational ICH advertisement, “Cinnamon wine with fragrance”, mainly demonstrating methods for cooking with sweet wine. In the video, the raw materials, processing crafts, and finished product are repeatedly shown, emphasizing the health benefits, lack of additives, and delicious taste of sweet wine (Figure 1). 

**Figure 2:**
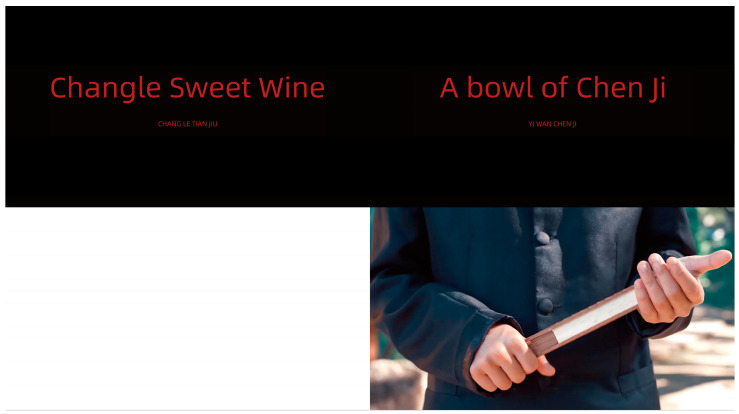
Ad 2: emotional advertisement entitled “Fortune-telling”.

Ad 2: An emotional advertisement, “Fortune-telling”, presenting the story of “corporate slaves” being redeemed. The main characters are young people, and the theme follows the issues that they face (Figure 2).

Eighty participants were randomly assigned to rate these two advertisements using a self-designed 5-item, 7-point scale. An independent sample *t*-test was used to detect the strength of the self-reference effect to distinguish the two types of advertisement [36]. The control test results showed that the total self-reference effect of the two types of advertising on the participants was significantly different, but the difference was small (Cohen’s d = 0.363), and self-reference was brought on more by the emotional advertisement (*t* = 3.065, *p* < 0.01). These results demonstrated that the control of both types of advertisement were effective.

#### 2.3.2. Experimental Design

1. Research Sample

In this experiment, 285 valid questionnaires were obtained from freshmen to seniors in Xiangtan University, of which 95 were men and 190 were women; the sample age range was from 18 to 25 years old. The experimental questionnaire was distributed online and filled in offline simultaneously.

2. Independent Variable Control

A single-factor design was used in the formal experiment. The independent variable is the type of advertising, with two categories: rational ad and emotional ad.

3. Dependent Variable Measurement

The dependent variables to be measured in the formal experiment are as follows: cognitive thinking response, emotional response, attitude towards advertisement, and purchasing attitude. An open questionnaire was designed for measuring the cognitive thinking response [37]. The emotional response was measured using an adjective 7-point rating scale, which includes negative and positive emotions composed of 20 emotional state adjectives that have generally been recognized previously in the literature [32]. Advertisement attitude was measured using a 4-item, 7-point rating scale [38]. Purchasing attitude was measured using a 7-point rating scale [39]. All the measurement questionnaires used in the formal experiment are included in the Supplementary Material.

4. Experimental Procedure

The formal experiment was divided into three phases. First, participants viewed advertising materials; second, they completed the questionnaire; and third, the retest phase was carried out, in which the dependent variable measured in the retest stage was brand recognition. The retest was conducted one week after the first stage of the formal experiment, and the questionnaires were sent to the formal experiment participants via the Internet. Brand recognition was tested using single-choice items.

### 2.4. Results

#### 2.4.1. Validity and Reliability Analysis of the Questionnaire

Structural validity is used to test the validity of the questionnaire. During the verification process, principal component analysis (PCA) and the varimax rotation method are adopted, while Cronbach’s α coefficient is used for reliability testing.

1. Cognitive Thinking Response Coding

This section was designed as open-ended questions. In this study, participants’ cognitive (thinking) responses were classified and coded. Based on previous research and the theoretical assumptions of this study, participants’ cognitive responses were categorized into three types [40]: (1) those involving their own past experiences, (2) those involving product attributes, and (3) those involving other aspects. Content involving personal experiences includes personal emotions, memories, etc.; content involving product attributes includes product functions, craftsmanship, raw materials, taste, etc.; and content involving other aspects includes the plot of the advertisement, shooting techniques, and other irrelevant content. Two statistics experts from Xiangtan University scored the responses, calculating the proportion of each participant’s three types of cognitive response in their total responses. To ensure the reliability of our coding results, we calculated the interrater reliability using Cohen’s Kappa. The Kappa values are as follows: involving personal experiences: *κ* = 0.77, involving other aspects: *κ* = 0.61, involving product attributes: *κ* = 0.80, and the inconsistent content was discussed and reconciled to reach consensus by two experts. The coding work was completed.

2. The Validity and Reliability of Each Scale

The validity and reliability of the emotional response scale, attitude towards the advertisement scale, and product attitude scale, impacted by ICH ad, were analyzed (see Table 1). According to the theoretical considerations, KMO and KMO validity analyses were tested first, and the results showed that both were very suitable for factor analysis; then, the exploratory factor analysis was carried out. Finally, we then conducted the reliability analysis. A comprehensive analysis showed that all of the above three scales have good validity and reliability.

#### 2.4.2. Hypothesis Testing

1. Examination of the Effect of the Two Types of ICH Ad on Emotional Responses, Advertisement Attitude, and Purchasing Attitude

For H1 and H2, the independent sample *t*-test was used to detect whether the emotional response, attitude towards the advertisement, and purchasing attitude that were stimulated by the two ICH ad is different.

(1) Emotional Response Examination

The emotional ICH ad elicited a stronger positive emotional response in participants (*t* = 3.497, *p* < 0.01), while there was no significant difference in the negative emotional response (*t* = −0.051, *p* = 0.960) between the two types of ICH ad. H1 was validated (see Table 2).

(2) Examination of Attitude Towards the Advertisements

There was no significant difference in the attitude among the participants who viewed the two types of ICH ad (*t* = 1.27, *p* = 0.205); the results indicate that, although the advertisement attitude score for emotional ICH ad was slightly higher than that for rational ICH ad, this difference was not statistically significant. Therefore, H2a was not supported (see Table 3).

(3) Purchasing Attitude Examination

There were obvious differences in viewers’ purchasing attitudes based on the two ICH ad (*t* = 2.27, *p* = 0.024), and the rational ICH ad viewers showed stronger buying intentions. H2b was not verified (see Table 4).

2. Examination of the Effect of the Two Types of Advertisement on Cognitive Thinking Response and Brand Recognition
(1) Cognitive Thinking Response Examination

Independent sample *t*-tests were used to examine the effect of the ICH ad types on cognitive thinking responses. The results showed that content involving other aspects was not significant (*t* = 1.292, *p* = 0.197). However, under the emotional ICH ad type, more participants thought about personal memory-related content (involving personal experiences, *t* = 3.033, *p* = 0.003), such as movies that they had seen or situations with family and friends. Under the rational ICH ad type, participants were significantly more likely to think about product attributes (*t* = −4.671, *p* < 0.001), such as raw material quality, manufacturing process, and taste (see Table 5). These results support H3a and H3b.

(2) Brand Recognition Examination

Participants who had watched the ICH ad one week earlier underwent a brand retest using a chi-square test. The results showed that the Chenji brand recognition accuracy rate for emotional ICH ad was 54.23%, while for rational ICH ad it was 36.36% (see Table 6). This indicates that emotional ICH ad significantly outperformed rational ICH ad in terms of brand recognition (*p* < 0.001), thus verifying H4.

### 2.5. Discussion 

Our results indicate that under the condition of rational ICH ad, participants think more about the product’s attributes and have a more positive purchasing attitude. This is likely because detailed product information and clear introductions of its functions help participants better understand a product’s actual uses and advantages. Conversely, under emotional ICH ad, participants experience stronger positive emotions and higher brand recognition accuracy, suggesting that emotional ad effectively captures viewers’ attention and create a lasting brand impression. Therefore, rational ICH ad is advantageous for recalling product attributes and enhancing purchasing attitudes, while emotional ICH ad excels in capturing attention, causing emotions, and improving brand recognition.

Due to the everyday and inexpensive nature of “Changle Sweet Rice Wine”, participants do not spend much time processing its product information; therefore, the straightforward product introduction in rational ICH ad can further stimulate purchasing motivation. As was found by Ching, Tong, and Chen [41], rational information prompts deeper cognitive processing, enhancing product understanding and recognition. However, the positive emotions and brand recognition elicited by emotional ICH ad can aid in building the ICH brand’s image. This aligns with Escalas’ view [42] that emotional ad enhances brand memory and emotional attitudes through emotional resonance.

Additionally, this study found that while emotional ICH ad prompts participants to reflect on personal experiences, they do not lead to more positive advertising and purchasing attitudes. This can be explained by Van den Bergh and Behrer’s research [43], which suggests that when advertisement-induced emotions do not align with an audience’s cognition or actual needs, cognitive dissonance occurs, preventing emotions from being translated into positive attitudes or behaviors. This dissonance may arise because the audience resonates with the emotional story in the ICH ad, but their actual needs or purchasing motivation are unmet, reducing the positive impact of emotions on attitudes towards advertisements and purchasing intentions.

Compared with general commercial advertisements, ICH ad not only aims to promote products but also carries the mission of preserving and promoting cultural heritage. Thus, ICH ad needs to balance showcasing product attributes and functions with conveying cultural connotations and emotional expressions. While most commercial advertisements focus on a product’s functionality and market competitiveness, ICH ad has a unique advantage in demonstrating cultural backgrounds and historical heritage, effectively transmitting cultural values and resonating with the audience [44]. Unlike general advertisements that primarily aim to attract immediate attention and drive purchasing decisions [45], emotional ICH ad creates lasting brand impressions by evoking emotional resonance [46,47]. However, the factors influencing an audience are complex and largely dependent on the ad’s content and form, dissemination channels, and the audience’s cultural background and values. Advertising messages need to be compelling enough to prompt deep cognitive processing. If an ICH ad’s content is too abstract or disconnected from the audience’s daily life, the audience may only engage in superficial processing without deeper consideration. Therefore, to achieve the true purpose of ICH ad, it is crucial to deeply understand the audience’s actual needs and expectations and how to effectively communicate the value and significance of ICH. This study explores the psychological effects of different types of ICH ad, enriching the research on advertisement appeals in the ICH field.

## 3. Study 2: Comparative Study on the Advantageous Effects of Emotional and Rational ICH Ad

### 3.1. Research Aim

In the preliminary field research, it was found that consumers have various needs when purchasing ICH products, with the primary purposes being own use and gifting. Understanding the classification of product use helps us understand consumers’ purchasing motivations and behaviors. Previous research supports the idea of advertising empathy, pointing out that advertising empathy is situational, occurring only under appropriate conditions, and closely related to the level of consumer involvement. In Study 1, the hypothesis that the ICH ad type affects an audience’s attitudes and purchasing attitudes was not proven. To further investigate the interactive effects of ICH ad types, different levels of situational involvement were included. Situational involvement is determined by the audience’s personal interests and importance in a specific purchasing context [48]. Consumers did, indeed, exhibit different psychological processes and choices when shopping for themselves versus shopping for others [49]. In this study, purchasing for oneself was set as high situational involvement, and purchasing gifts for others was set as low situational involvement. With this distinction, we aimed to explore how different levels of situational involvement affect an audience’s attitudes and purchasing attitudes after watching ICH ad.

### 3.2. Research Hypotheses

According to the elaboration likelihood model (ELM) [50], the way that audiences process advertisement information is influenced by their level of situational involvement, a widely recognized perspective. Under high involvement, consumers tend to process advertisement information through the central route, meaning that they carefully analyze all the information and arguments presented in the advertisement [51] to reduce purchasing risks. Conversely, under low involvement, consumers tend to process information through the peripheral route, being more easily influenced by emotions and intuition. Emotional ad is more effective in such cases. Emotional ad can evoke stronger emotional resonance and positive attitudes under low involvement [52] and thus more effectively influence consumers’ purchasing attitudes and behaviors [53]. Therefore, situational involvement plays a key role in determining the effectiveness of ICH ad. Based on the above analysis, this study hypothesizes the following:

**H5a.**  *The type of ICH ad and situational involvement have a significant interactive effect on the attitudes towards an advertisement. Under high situational involvement, no significant difference in attitudes is elicited by emotional and rational ICH ad. Under low situational involvement, emotional ICH ad elicits significantly better attitudes than rational ICH ad does.*

**H5b.**  *The type of ICH ad and situational involvement have a significant interactive effect on purchasing attitudes. Under high situational involvement, no significant difference in purchasing attitudes is elicited by emotional and rational ICH ad. Under low situational involvement, emotional ICH ad elicits significantly better purchasing attitudes than rational ICH ad does.*

### 3.3. Research Methods

#### 3.3.1. Pretest: Control Test of Situational Involvement Instruction 

This study controlled the level of situational involvement through instructional scenarios. The high-situational-involvement instruction scenario is described as follows: “Imagine you are purchasing a high-quality bottle of Changle Sweet Rice Wine for yourself. This sweet wine is an intangible cultural heritage product, representing traditional brewing techniques and a unique cultural background. You highly value this purchase and want to choose a sweet wine that best suits your taste and needs. You will carefully read product information, understand the brewing process, taste different brands, and finally make a well-considered purchase decision”. The low-situational-involvement instruction scenario was described as follows: “Imagine you are purchasing a high-quality bottle of Changle Sweet Rice Wine as a gift for someone else. This sweet wine is an intangible cultural heritage product, representing traditional brewing techniques and a unique cultural background. You highly value this purchase and want to choose a sweet wine that best suits the recipient’s taste and needs. You will briefly read product information, understand the basic brewing process, taste different brands, and quickly make a purchase decision”.

Two hundred participants were randomly selected to rate the level of involvement induced by the two different instructions using a self-designed 5-item, 7-point scale. An independent sample *t*-test was used to detect the differences. The results showed that the mean standardized total score for the low-situational-involvement group was 2.91 (*SD* = 1.24), and that for the high-situational-involvement group it was 4.85 (*SD* = 1.23). The independent sample *t*-test result was *t* = −11.09, *p* < 0.0001, indicating a significant difference in involvement levels. This further confirmed the effectiveness of the design of the instructions in this study.

#### 3.3.2. Experimental Design

1. Research Sample

In this experiment, 400 valid questionnaires were obtained from freshmen to seniors at Xiangtan University, of which 194 were men and 206 were women; the sample age range was from 18 to 25 years old. The experimental questionnaire was distributed online via the Wenjuanxing platform and filled in offline simultaneously.

2. Independent Variable Control

The experiment adopted a mixed-factor design, utilizing advertisement type (rational ad and emotional ad) and situational involvement (high situational involvement and low situational involvement) as independent variables.

3. Dependent Variable Measurement

The dependent variables to be measured in the experiment included attitude towards the advertisement and purchasing attitude. The questionnaire design was the same as in the first stage.

4. Experimental Procedure

The formal experiment was divided into three phases: first, a live broadcast of the situational involvement instruction; second, participants viewed the advertising materials; and third, they completed the questionnaire.

### 3.4. Results

To verify hypotheses H5a and H5b, a two-factor analysis of variance (ANOVA) was first conducted to explore the interactive effects of advertisement type and situational involvement on the attitude towards the advertisement and purchasing attitude. To further explore these interactive effects, a Tukey HSD post hoc test was also performed.

1. On the Attitude towards the Ad

To investigate the impact of the advertisement type and situational involvement on the attitude towards the advertisement, a 2 (advertisement type: emotional ad, rational ad) × 2 (situational involvement: high, low) within-subjects factorial ANOVA was conducted. The results showed that the main effect of advertisement type on the attitude towards the advertisement was not significant: *F* = 3.804, *df* = 1, *MS* = 125.44, *p* = 0.052, *η*^2^ = 0.0094. The main effect of situational involvement on the attitude towards the advertisement was significant: *F* = 7.100, *df* = 1, *MS* = 234.09, *p =* 0.008, *η*^2^ = 0.0177. The interactive effect of advertisement type and situational involvement on the attitude towards the advertisement was also significant: *F =* 4.514, *df* = 1, *MS* = 148.84, *p* = 0.034, *η*^2^ = 0.0112.

Combined with Table 7, it can be seen that, under low situational involvement, emotional ICH ad (*M* = 18.52) elicited more positive attitudes compared with rational ICH ad (*M* = 16.18). However, under high situational involvement there was no significant difference in the attitudes elicited by the two types of ICH ad (*M* = 15.74, *M* = 15.77). Further a simple effects analysis also showed that under low situational involvement, emotional ad elicited significantly more positive attitudes compared with rational ad (*t* = 2.882, *MD* = 2.340, *p* = 0.004). This indicates that when situational involvement is low, emotional ad is more effective in enhancing viewers’ attitudes. Under high situational involvement, there was no significant difference in the attitudes elicited by emotional and rational ad (*t* = −0.123, *MD* = −0.100, *p* = 0.902). This indicates that when the situational involvement is high, emotional and rational ad has no significant difference in their impact on advertisement attitudes. H5a is verified.

2. On Purchasing Attitude

To investigate the impact of the ICH ad type and situational involvement on purchasing attitude, a 2 (advertisement type: emotional ICH ad, rational ICH ad) × 2 (situational involvement: high, low) within-subjects factorial ANOVA was conducted. The results showed that the main effect of the ICH ad type on purchasing attitude was not significant: *F* = 0.484, *df* = 1, *MS* = 1.21, *p* = 0.487, *η*^2^ = 0.0012. The main effect of situational involvement on purchasing attitude was significant: *F* = 4.622, *df* = 1, *MS* = 11.56, *p* = 0.032, *η*^2^ = 0.0111. Additionally, the interactive effect of the ICH ad type and situational involvement on purchasing attitude was significant: *F* = 13.451, *df* = 1, *MS* = 33.64, *p* < 0.001, *η*^2^ = 0.0324. The specific data are presented below.

Combined with Table 8, it can be seen that, under low situational involvement, emotional ICH ad (*M* = 5.71) elicited higher purchasing attitudes compared with rational ICH ad (*M* = 5.02). However, under high situational involvement, emotional ICH ad (*M =* 4.79) elicited lower purchasing attitudes compared with rational ICH ad (*M* = 5.26). A further simple effects analysis also showed that, under low situational involvement, emotional ICH ad elicited significantly higher purchasing attitudes compared with rational ICH ad (*t* = 3.085, *MD* = 0.690, *p* = 0.002). This indicates that, when the situational involvement is low, emotional ICH ad is more effective in enhancing purchasing attitudes. Under high situational involvement, emotional ICH ad elicited significantly lower purchasing attitudes compared with rational ICH ad (*t* = −2.102, *MD* = −0.470, *p* = 0.036). This indicates that, when the situational involvement is high, rational ICH ad is more effective in enhancing purchasing attitudes. H5b is partially verified.

### 3.5. Discussion 

Our results show that under low situational involvement, emotional ICH ad elicits more positive attitudes towards an advertisement, while rational ICH ad stimulates more positive purchasing attitudes. This is because, with low involvement, participants pay less attention to an advertisement’s content and are more easily attracted by superficial factors, resulting in a favorable view of the advertisement itself. However, when it comes to actual purchasing, the specific product information and introductions of its functions in rational ICH ad is more persuasive and enhance purchasing intent. This phenomenon is supported by the research by Akbari and Otamendi [35,46].

Under high situational involvement, there is no significant difference in the impact of the two types of ICH ad on viewers’ attitude towards an advertisement. With high situational involvement, participants pay more attention to and engage more with the advertisement, leading to a more rational and comprehensive evaluation of the information. Therefore, neither advertisement type significantly affects advertisement attitudes under high situational involvement. However, there is a significant difference in their impact on purchasing attitudes, with rational ICH ad being more effective in enhancing purchasing attitudes. This indicates that, under high situational involvement, participants focus more on the product information provided in the advertisement, revealing that consumers may rely more on factors such as taste, quality, or craftsmanship to make purchasing decisions.

This result reveals that, under high situational involvement, rational ICH ad is more effective in enhancing purchasing attitudes. This suggests that consumers with high situational involvement may depend more on the inherent attributes of ICH products to make purchasing decisions. This finding is consistent with previous studies, which show that the detailed product information in rational ad can be more convincing when consumers are highly involved in the purchasing decision process. In contrast, under low situational involvement, emotional appeals are more effective in capturing attention and creating a positive impression of the advertisement itself.

It is worth noting that ICH ad under high situational involvement exhibit different characteristics from those of regular advertising. The effectiveness of regular advertising under high situational involvement mainly relies on the persuasiveness of the information. In contrast, ICH ad not only needs to provide detailed product information but also convey rich cultural connotations and emotional elements. This indicates that ICH ad under high situational involvement need to strike a balance between rationality and emotion to meet the multilevel needs of consumers.

## 4. Conclusions

This study uses the dietary ICH product “Changle Sweet Wine” as its research material and systematically explores the psychological effects of ICH ad through a step-by-step research methodology. This approach helps us gain a comprehensive understanding of the different impacts of rational and emotional ad.

Firstly, Study 1 clearly lists five measurement indicators (cognitive thinking, emotional response, attitude towards ad, purchasing attitude, and brand recognition), providing a clear framework for evaluating the effectiveness of ICH ad. It was found that emotional ICH ad is more likely to evoke audience memories, enhance positive emotions, and improve brand recognition, while rational ICH ad is more likely to draw attention to the product’s attributes. However, Study 1 also revealed that the ICH ad type does not significantly affect viewers’ attitudes towards an advertisement, and rational ICH ad is more likely to stimulate higher purchasing attitudes.

In Study 2, the key factor of situational involvement was introduced, as it can influence an audience’s cognitive and emotional responses to advertisements and products, thereby affecting their attitudes towards the advertisement and purchasing attitudes. It was found that emotional ICH ad is more effective in enhancing both viewers’ attitudes towards the advertisement and purchasing attitudes under low situational involvement. However, rational ICH ad does not have an advantage in enhancing the attitudes towards the advertisement under different situational involvement levels, but they do elicit stronger purchasing attitudes under high situational involvement. Rational ICH ad consistently stimulates higher purchasing attitudes, regardless of situational involvement.

This study compares the respective advantages of rational and emotional ad from multiple aspects, offering ICH practitioners better choices for advertisement types based on different situations. When designing advertisement strategies, ICH practitioners should fully consider the interaction between situational involvement and advertisement type, selecting appropriate advertisement formats to better meet the needs and expectations of their target audience and thus achieving market promotion and brand building for ICH products.

For example, the gift-pack product of Chenji Changle Sweet Wine should be featured in emotional ad that emphasizes ICH-related culture, history, and personal experiences, and these advertisements should be placed in gift shops, airports, hospitals, and on social media platforms with keywords related to ICH-related culture, gifts, sentiments, and memories. Conversely, the basic-pack product should feature rational ad that highlights the product’s functionality, quality, and craftsmanship, and these advertisements should be placed in supermarkets, convenience stores, markets, and on social media platforms with keywords related to ICH craftsmanship, flavor, and rice aroma. Advertisements should be created and placed based on the different usage needs of consumers.

The results of this study not only fill the research gap in evaluating the psychological effects of ICH ad types but also provide empirical support. Additionally, they offer new perspectives for the modern continuation and development of ICH.

## 5. Limitations and Future Research Directions

The participants of this study were college students, indicating a lack of sample diversity. Consequently, the research results have certain limitations. Future studies should consider a broader sample to enhance representativeness. Additionally, the research material was limited to a dietary ICH brand, which might affect the generalizability of the findings. Future research could explore advertisements for other types of ICH products, such as handicrafts and traditional performing arts.

Furthermore, the advertisements in this study were not of a high production quality, and there were shortcomings in the plot design and visual language, which could have impacted the results. Future research should address these issues to improve the quality of the advertisements.

Moreover, there are some limitations in this study. Future research could explore the effects of ICH ad in different cultural contexts to understand how cultural differences influence the attitudes towards an advertisement and purchasing intentions. It could also investigate the impact of ICH ad on brand loyalty and long-term purchasing behavior to understand their role in long-term marketing strategies.

## Figures and Tables

**Table 1:** Validity and reliability analysis of scales.

	Validity				Reliability
	KMO	Bartlett’s Test of Sphericity			Cronbach’s α Coefficient
		Approximate Chi-Square	df	*p*	
Emotional Response Scale	0.921	6184.921	190	0.000	0.925
Advertisement Attitude Scale	0.829	1265.157	6	0.000	0.98

**Table 2:** Independent sample *t*-test of the emotional responses triggered by the two types of advertisement.

	Advertisement Type	Sample Capacity	Average Value	Standard Deviation	*t*	*p*	Cohen’s d Value
Positive emotional response	Rational Ad	143	3.984	1.454	3.497	0.001	0.414
	Emotional Ad	142	3.329	1.699			
Negative emotional response	Rational Ad	143	3.983	1.454	−0.051	0.960	0.006
	Emotional Ad	142	3.329	1.699			

**Table 3:** Independent sample *t*-test of advertising attitude.

	Advertisement Type	Sample Capacity	Average Value	Standard Deviation	*t*	*p*	Cohen’s d Value
Advertising attitude	Rational Ad	143	2.383	1.586	1.27	0.205	0.15
	Emotional Ad	142	2.632	1.724			

**Table 4:** Independent sample *t*-test of purchasing attitude.

	Advertisement Type	Sample Capacity	Average Value	Standard Deviation	*t*	*p*	Cohen’s d Value
Purchasing attitude	Rational Ad	143	4.175	1.646	2.276	0.024	0.27
	Emotional Ad	142	3.718	1.74			

**Table 5:** Independent sample *t*-tests for cognitive thinking responses.

	Advertisement Type	Sample Capacity	Average Value	Standard Deviation	*t*	*p*	Cohen’s d Value
Involving Personal Experiences	Rational Ad	143	0.26	0.38	3.033	0.003	0.360
	Emotional Ad	142	0.41	0.46			
Involving Other Aspects	Rational Ad	143	0.35	0.45	1.292	0.197	0.153
	Emotional Ad	142	0.42	0.46			
Involving Product Attributes	Rational Ad	143	0.40	0.44	−4.671	0.000	0.553
	Emotional Ad	142	0.17	0.36			

**Table 6:** Chi-square test for brand recognition under two types of ICH ad.

Brand	Advertisement Type (%)		Total	χ^2^	*p*
	Rational Ad	Emotional Ad			
Yiwan Xuji	23 (16.08)	26 (18.31)	49 (17.19)	31.331	0.000
Jideli	9 (6.29)	6 (4.23)	15 (5.26)		
Chenji	52 (36.36)	77 (54.23)	129 (45.26)		
Huatiangangzi	17 (11.89)	24 (16.90)	41 (14.39)		
Wowo	3 (2.10)	3 (2.11)	6 (2.11)		
Mipopo	20 (13.99)	2 (1.41)	22 (7.72)		
Rishiji	19 (13.29)	4 (2.82)	23 (8.07)		
Total	143	142	285		

**Table 7:** Analysis of attitudes towards advertisements under different ICH ad types and degrees of situational involvement.

	*M*	*SD*	*N*
Low Situational Involvement × Emotional Ad	18.52	5.27	100
Low Situational Involvement × Rational Ad	16.18	5.90	100
High Situational Involvement × Emotional Ad	15.77	5.33	100
High Situational Involvement × Rational Ad	15.87	6.40	100

**Table 8:** Analysis of purchasing attitude under different ICH ad types and degrees of situational involvement.

	*M*	*SD*	*N*
Low Situational Involvement × Emotional Ad	5.71	1.45	100
Low Situational Involvement × Rational Ad	5.02	1.61	100
High Situational Involvement × Emotional Ad	4.79	1.74	100
High Situational Involvement × Rational Ad	5.26	1.50	100

## Data Availability

The datasets generated and/or analyzed during the current study are available from the corresponding author on reasonable request.

## Supplementary Materials

The following supporting information can be downloaded at https://www.mdpi.com/article/10.3390/bs14070623/s1.
