# Supplementary material for: Study on the Psychological Effects of Intangible Cultural Heritage Advertising with Different Degrees of Situational Involvement
Source: Behav Sci (Basel). 2024 Jul 22;14(7):623. doi: 10.3390/bs14070623 (PMC11274228; doi:10.3390/bs14070623)
Supplement: Supplementary file 1 [file behavsci-14-00623-s001.zip › Attachment 1 Pre-test_Questionnaire.pdf]

Attachment 1: Pre-test Questionnaire

1. Your gender: [Single choice question] *

–○ Male

–○ Female

I. Advertisement Screening Questionnaire

Please watch the following video advertisements and determine which type of advertisement it is:
(Rational advertisements present factual evidence or make comparisons, usually showcasing product features, uses, methods of use, and other factual information about the product. Emotional advertisements appeal to consumers' emotions or emotional responses, such as humor, empathy, and memories, conveying the added value or emotional satisfaction brought by the product.) [Single choice question]

Rational advertisement	1	2	3	4	5	6	7	Emotional advertisement
	○	○	○	○	○	○	○	
	○	○	○	○	○	○	○	
	○	○	○	○	○	○	○	
	○	○	○	○	○	○	○	
	○	○	○	○	○	○	○	
	○	○	○	○	○	○	○	
	○	○	○	○	○	○	○	

Please watch the following video advertisements and determine which type of advertisement it is:
(Rational advertisements present factual evidence or make comparisons, usually showcasing product features, uses, methods of use, and other factual information about the product. Emotional advertisements appeal to consumers' emotions or emotional responses, such as humor, empathy, and memories, conveying the added value or emotional satisfaction brought by the product.) [Single choice question]

Rational advertisement	1	2	3	4	5	6	7	Emotional advertisement
	○	○	○	○	○	○	○	
	○	○	○	○	○	○	○	
	○	○	○	○	○	○	○	
	○	○	○	○	○	○	○	
	○	○	○	○	○	○	○	
	○	○	○	○	○	○	○	
	○	○	○	○	○	○	○	

II. Scenario Involvement Control Questionnaire

1. To ensure the effectiveness of scenario involvement manipulation, we have designed a questionnaire to assess participants' involvement levels in different scenarios. Please answer the following questions based on your true feelings.

(1) Scenario Description: Imagine you are buying a high-quality bottle of Changle sweet wine for yourself. This sweet wine is an intangible cultural heritage, representing traditional brewing techniques and unique cultural background. You highly value this purchase and hope to choose the sweet wine that best suits your taste and needs. You will carefully read product information, understand brewing techniques, taste different brands, and finally make a well-considered purchase decision.

(2) Instructions: In the following task, please carefully consider your evaluation and choice of Changle sweet wine based on the provided information. Imagine you are buying this sweet wine for yourself, and your choice will directly affect your satisfaction. (Please indicate your level of agreement with the following descriptions: 1 Completely agree; 7 Completely disagree) [Matrix scale question] *

	1	2	3	4	5	6	7
In this decision process, I feel this decision is very important.	○	○	○	○	○	○	○
I spent a lot of time and effort considering this decision.	○	○	○	○	○	○	○
I care deeply about the decision I made.	○	○	○	○	○	○	○

I feel this decision will have a significant impact on me.	○	○	○	○	○	○	○
When making this decision, I will carefully weigh various options.	○	○	○	○	○	○	○

2. To ensure the effectiveness of scenario involvement manipulation, we have designed a questionnaire to assess participants' involvement levels in different scenarios. Please answer the following questions based on your true feelings.

(1) Scenario Description: Imagine you are buying a bottle of Changle sweet wine as a gift for a colleague. This sweet wine is an intangible cultural heritage, representing traditional brewing techniques and unique cultural background. Although you want to choose a good gift, this decision is not important to you personally. You will not spend much time and effort researching detailed information about each product, just quickly selecting a sweet wine that looks good.

(2) Instructions: In the following task, please quickly choose the Changle sweet wine that you think is suitable as a gift based on the provided information. Imagine you are buying this sweet wine for a colleague, and this choice is not important to you personally. (Please indicate your level of agreement with the following descriptions: 1 Completely agree; 7 Completely disagree) [Matrix scale question] *

	1	2	3	4	5	6	7
In this decision process, I feel this decision is very important.	○	○	○	○	○	○	○
I spent a lot of time and effort considering this decision.	○	○	○	○	○	○	○
I care deeply about the decision I made.	○	○	○	○	○	○	○
I feel this decision will have a significant impact on me.	○	○	○	○	○	○	○
When making this decision, I will carefully weigh various options.	○	○	○	○	○	○	○
