# Supplementary material for: Study on the Psychological Effects of Intangible Cultural Heritage Advertising with Different Degrees of Situational Involvement
Source: Behav Sci (Basel). 2024 Jul 22;14(7):623. doi: 10.3390/bs14070623 (PMC11274228; doi:10.3390/bs14070623)
Supplement: Supplementary file 1 [file behavsci-14-00623-s001.zip › Attachment 2 First Step-Formal_Experiment_Questionnaire.pdf]

Formal Experiment Measurement Questionnaire

https://haokan.baidu.com/v?vid=4165559206300510173&pd=pcshare&hkRelaunch=p1%3Dpc%26p2%3Dvideoland%26p3%3Dshare_input

1. What emotions did you feel while watching the above advertisement? (Please indicate your level of agreement with the following emotions, 1 completely disagree, 7 completely agree)

	1	2	3	4	5	6	7
Happy	○	○	○	○	○	○	○
Kind	○	○	○	○	○	○	○
Embarrassed	○	○	○	○	○	○	○
Troubled	○	○	○	○	○	○	○
Disappointed	○	○	○	○	○	○	○
Excited	○	○	○	○	○	○	○
Angry	○	○	○	○	○	○	○
Happy	○	○	○	○	○	○	○
Afraid	○	○	○	○	○	○	○
Satisfied	○	○	○	○	○	○	○
Regretful	○	○	○	○	○	○	○
Glad	○	○	○	○	○	○	○
Surprised	○	○	○	○	○	○	○
Sad	○	○	○	○	○	○	○
Nervous	○	○	○	○	○	○	○
Loving	○	○	○	○	○	○	○
Warm	○	○	○	○	○	○	○
Lost	○	○	○	○	○	○	○

Grateful	○	○	○	○	○	○	○
Hopeful	○	○	○	○	○	○	○

2. Do you believe the product in the advertisement has the following attributes:

	1	2	3	4	5	6	7
Delicate taste	○	○	○	○	○	○	○
Smooth and mellow	○	○	○	○	○	○	○
Delicious and sweet	○	○	○	○	○	○	○
Traditional craftsmanship	○	○	○	○	○	○	○
Long history	○	○	○	○	○	○	○
Nutritious and healthy	○	○	○	○	○	○	○

3. Do you think the following attributes of the product are important:

	1	2	3	4	5	6	7
Delicate taste	○	○	○	○	○	○	○
Smooth and mellow	○	○	○	○	○	○	○
Delicious and sweet	○	○	○	○	○	○	○
Traditional craftsmanship	○	○	○	○	○	○	○
Long history	○	○	○	○	○	○	○
Nutritious and healthy	○	○	○	○	○	○	○

4. Do you think the product in the advertisement is:

	1	2	3	4	5	6	7

Effective	○	○	○	○	○	○	○
Beneficial	○	○	○	○	○	○	○
Functional	○	○	○	○	○	○	○
Necessary	○	○	○	○	○	○	○
Practical	○	○	○	○	○	○	○
Interesting	○	○	○	○	○	○	○
Pleasant	○	○	○	○	○	○	○
Exciting	○	○	○	○	○	○	○
Enjoyable	○	○	○	○	○	○	○
Good	○	○	○	○	○	○	○
Satisfactory	○	○	○	○	○	○	○
Attractive	○	○	○	○	○	○	○
Likeable	○	○	○	○	○	○	○

5. Do you think the advertisement is:

	1	2	3	4	5	6	7
Good	○	○	○	○	○	○	○
Likeable	○	○	○	○	○	○	○
Positive	○	○	○	○	○	○	○
Pleasant	○	○	○	○	○	○	○

6. Please rate the thoughts that came to your mind while watching the ad. Please indicate your level of agreement with the following descriptions:

	1	2	3	4	5	6	7
I can relate myself to the ad	○	○	○	○	○	○	○
I think the ad is related to	○	○	○	○	○	○	○

my own experiences							
I am thinking about the sweet wine drinks I had before	○	○	○	○	○	○	○
I am thinking about the brewing process of sweet wine	○	○	○	○	○	○	○
The ad reminds me of my own drinking experiences	○	○	○	○	○	○	○

7. If the product is available on the market, are you likely to buy it?

	1	2	3	4	5	6	7
Definitely	○	○	○	○	○	○	○
2	○	○	○	○	○	○	○
3	○	○	○	○	○	○	○
4	○	○	○	○	○	○	○
5	○	○	○	○	○	○	○
6	○	○	○	○	○	○	○
Definitely not	○	○	○	○	○	○	○

Personal Information (This information is for data statistics only and will be kept confidential)

Your gender:

○ Male

○ Female

Your age group:

○ Under 18

○ 18-25

○ 26-30

○ 31-40

○ 41-50

○ 51-60

○ Over 60
