# Supplementary material for: Study on the Psychological Effects of Intangible Cultural Heritage Advertising with Different Degrees of Situational Involvement
Source: Behav Sci (Basel). 2024 Jul 22;14(7):623. doi: 10.3390/bs14070623 (PMC11274228; doi:10.3390/bs14070623)
Supplement: Supplementary file 1 [file behavsci-14-00623-s001.zip › Attachment 2 Second Step-Formal_Experiment_Questionnaire.pdf]

Attachment 2

Formal Experiment Measurement Questionnaire 1

Scenario Description:

Imagine you are buying a bottle of Changle Sweet Wine as a gift for a colleague. This sweet wine is an intangible cultural heritage, representing traditional brewing techniques and a unique cultural background. Although you want to choose a nice gift, this decision is not personally important to you. You won't spend much time and effort researching the product details, just quickly select a bottle of sweet wine that looks good.

Instructions:

In the following tasks, please quickly choose the Changle Sweet Wine you think is suitable as a gift based on the provided information. Imagine you are buying this sweet wine for a colleague, and this choice is not personally important to you.

Today, you will come across an advertisement for sweet wine again, and you need to obtain relevant information from the ad. Please watch the video advertisement below.

<LINK>
https://haokan.baidu.com/v?vid=4165559206300510173&pd=pcshare&hkRelaunch=p1%3Dpc%26p2%3Dvideoland%26p3%3Dshare_input

1. What emotions did you feel while watching the above advertisement? (Please indicate your level of agreement with the following emotions, 1 completely disagree, 7 completely agree)

	1	2	3	4	5	6	7
Happy	○	○	○	○	○	○	○
Kind	○	○	○	○	○	○	○
Embarrassed	○	○	○	○	○	○	○
Troubled	○	○	○	○	○	○	○
Disappointed	○	○	○	○	○	○	○
Excited	○	○	○	○	○	○	○
Angry	○	○	○	○	○	○	○
Happy	○	○	○	○	○	○	○

Afraid	○	○	○	○	○	○	○
Satisfied	○	○	○	○	○	○	○
Regretful	○	○	○	○	○	○	○
Glad	○	○	○	○	○	○	○
Surprised	○	○	○	○	○	○	○
Sad	○	○	○	○	○	○	○
Nervous	○	○	○	○	○	○	○
Loving	○	○	○	○	○	○	○
Warm	○	○	○	○	○	○	○
Lost	○	○	○	○	○	○	○
Grateful	○	○	○	○	○	○	○
Hopeful	○	○	○	○	○	○	○

2. Do you believe the product in the advertisement has the following attributes:

	1	2	3	4	5	6	7
Delicate taste	○	○	○	○	○	○	○
Smooth and mellow	○	○	○	○	○	○	○
Delicious and sweet	○	○	○	○	○	○	○
Traditional craftsmanship	○	○	○	○	○	○	○
Long history	○	○	○	○	○	○	○
Nutritious and healthy	○	○	○	○	○	○	○

3. Do you think the following attributes of the product are important:

	1	2	3	4	5	6	7
Delicate taste	○	○	○	○	○	○	○

Smooth and mellow	○	○	○	○	○	○	○
Delicious and sweet	○	○	○	○	○	○	○
Traditional craftsmanship	○	○	○	○	○	○	○
Long history	○	○	○	○	○	○	○
Nutritious and healthy	○	○	○	○	○	○	○

4. Do you think the product in the advertisement is:

	1	2	3	4	5	6	7
Effective	○	○	○	○	○	○	○
Beneficial	○	○	○	○	○	○	○
Functional	○	○	○	○	○	○	○
Necessary	○	○	○	○	○	○	○
Practical	○	○	○	○	○	○	○
Interesting	○	○	○	○	○	○	○
Pleasant	○	○	○	○	○	○	○
Exciting	○	○	○	○	○	○	○
Enjoyable	○	○	○	○	○	○	○
Good	○	○	○	○	○	○	○
Satisfactory	○	○	○	○	○	○	○
Attractive	○	○	○	○	○	○	○
Likeable	○	○	○	○	○	○	○

5. Do you think the advertisement is:

	1	2	3	4	5	6	7
Good	○	○	○	○	○	○	○

Likeable	○	○	○	○	○	○	○
Positive	○	○	○	○	○	○	○
Pleasant	○	○	○	○	○	○	○

6. Please rate the thoughts that came to your mind while watching the ad. Please indicate your level of agreement with the following descriptions:

	1	2	3	4	5	6	7
I can relate myself to the ad	○	○	○	○	○	○	○
I think the ad is related to my own experiences	○	○	○	○	○	○	○
I am thinking about the sweet wine drinks I had before	○	○	○	○	○	○	○
I am thinking about the brewing process of sweet wine	○	○	○	○	○	○	○
The ad reminds me of my own drinking experiences	○	○	○	○	○	○	○

7. If the product is available on the market, are you likely to buy it?

Definitely	1	2	3	4	5	6	7	Definitely not
	○	○	○	○	○	○	○	
	○	○	○	○	○	○	○	
	○	○	○	○	○	○	○	
	○	○	○	○	○	○	○	
	○	○	○	○	○	○	○	
	○	○	○	○	○	○	○	
	○	○	○	○	○	○	○	

Personal Information (This information is for data statistics only and will be kept confidential)

Your gender:

○ Male

○ Female

Your age group:

○ Under 18

○ 18-25

○ 26-30

○ 31-40

○ 41-50

○ 51-60

○ Over 60

Formal Experiment Measurement Questionnaire 2

Scenario Description:

Imagine you are buying a bottle of high-quality Changle Sweet Wine for yourself. This sweet wine is an intangible cultural heritage, representing traditional brewing techniques and a unique cultural background. You highly value this purchase and hope to choose the sweet wine that best suits your taste and needs. You will carefully read product information, understand the brewing process, taste different brands, and ultimately make a well-considered purchase decision.

Instructions:

In the following tasks, please carefully consider your evaluation and choice of Changle Sweet Wine based on the provided information. Imagine you are buying this sweet wine for yourself, and your choice will directly affect your satisfaction.

Today, you will come across an advertisement for sweet wine again, and you need to obtain relevant information from the ad. Please watch the video advertisement below.

<LINK>
https://haokan.baidu.com/v?vid=4165559206300510173&pd=pcshare&hkRelaunch=p1%3Dpc%26p2%3Dvideoland%26p3%3Dshare_input

1. What emotions did you feel while watching the above advertisement? (Please indicate your level of agreement with the following emotions, 1 completely disagree, 7 completely agree)

	1	2	3	4	5	6	7
Happy	○	○	○	○	○	○	○
Kind	○	○	○	○	○	○	○
Embarrassed	○	○	○	○	○	○	○
Troubled	○	○	○	○	○	○	○
Disappointed	○	○	○	○	○	○	○
Excited	○	○	○	○	○	○	○
Angry	○	○	○	○	○	○	○
Happy	○	○	○	○	○	○	○
Afraid	○	○	○	○	○	○	○
Satisfied	○	○	○	○	○	○	○

Regretful	○	○	○	○	○	○	○
Glad	○	○	○	○	○	○	○
Surprised	○	○	○	○	○	○	○
Sad	○	○	○	○	○	○	○
Nervous	○	○	○	○	○	○	○
Loving	○	○	○	○	○	○	○
Warm	○	○	○	○	○	○	○
Lost	○	○	○	○	○	○	○
Grateful	○	○	○	○	○	○	○
Hopeful	○	○	○	○	○	○	○

2. Do you believe the product in the advertisement has the following attributes:

	1	2	3	4	5	6	7
Delicate taste	○	○	○	○	○	○	○
Smooth and mellow	○	○	○	○	○	○	○
Delicious and sweet	○	○	○	○	○	○	○
Traditional craftsmanship	○	○	○	○	○	○	○
Long history	○	○	○	○	○	○	○
Nutritious and healthy	○	○	○	○	○	○	○

3. Do you think the following attributes of the product are important:

	1	2	3	4	5	6	7
Delicate taste	○	○	○	○	○	○	○
Smooth and mellow	○	○	○	○	○	○	○

Delicious and sweet	○	○	○	○	○	○	○
Traditional craftsmanship	○	○	○	○	○	○	○
Long history	○	○	○	○	○	○	○
Nutritious and healthy	○	○	○	○	○	○	○

4. Do you think the product in the advertisement is:

	1	2	3	4	5	6	7
Effective	○	○	○	○	○	○	○
Beneficial	○	○	○	○	○	○	○
Functional	○	○	○	○	○	○	○
Necessary	○	○	○	○	○	○	○
Practical	○	○	○	○	○	○	○
Interesting	○	○	○	○	○	○	○
Pleasant	○	○	○	○	○	○	○
Exciting	○	○	○	○	○	○	○
Enjoyable	○	○	○	○	○	○	○
Good	○	○	○	○	○	○	○
Satisfactory	○	○	○	○	○	○	○
Attractive	○	○	○	○	○	○	○
Likeable	○	○	○	○	○	○	○

5. Do you think the advertisement is:

	1	2	3	4	5	6	7
Good	○	○	○	○	○	○	○
Likeable	○	○	○	○	○	○	○

Positive	○	○	○	○	○	○	○
Pleasant	○	○	○	○	○	○	○

6. Please rate the thoughts that came to your mind while watching the ad. Please indicate your level of agreement with the following descriptions:

	1	2	3	4	5	6	7
I can relate myself to the ad	○	○	○	○	○	○	○
I think the ad is related to my own experiences	○	○	○	○	○	○	○
I am thinking about the sweet wine drinks I had before	○	○	○	○	○	○	○
I am thinking about the brewing process of sweet wine	○	○	○	○	○	○	○
The ad reminds me of my own drinking experiences	○	○	○	○	○	○	○

7. If the product is available on the market, are you likely to buy it?

Definitely	1	2	3	4	5	6	7	Definitely not
	○	○	○	○	○	○	○	
	○	○	○	○	○	○	○	
	○	○	○	○	○	○	○	
	○	○	○	○	○	○	○	
	○	○	○	○	○	○	○	
	○	○	○	○	○	○	○	
	○	○	○	○	○	○	○	

Personal Information (This information is for data statistics only and will be kept confidential)

Your gender:

○ Male

○ Female

Your age group:

○ Under 18

○ 18-25

○ 26-30

○ 31-40

○ 41-50

○ 51-60

○ Over 60

Formal Experiment Measurement Questionnaire 3

Scenario Description:

Imagine you are buying a bottle of Changle Sweet Wine as a gift for a colleague. This sweet wine is an intangible cultural heritage, representing traditional brewing techniques and a unique cultural background. Although you want to choose a nice gift, this decision is not personally important to you. You won't spend much time and effort researching the product details, just quickly select a bottle of sweet wine that looks good.

Instructions:

In the following tasks, please quickly choose the Changle Sweet Wine you think is suitable as a gift based on the provided information. Imagine you are buying this sweet wine for a colleague, and this choice is not personally important to you.

Today, you will come across an advertisement for sweet wine again, and you need to obtain relevant information from the ad. Please watch the video advertisement below.

<LINK>
https://haokan.baidu.com/v?vid=4481420460012793311&pd=pcshare&hkRelaunch=p1%3Dpc%26p2%3Dvideoland%26p3%3Dshare_input

1. What emotions did you feel while watching the above advertisement? (Please indicate your level of agreement with the following emotions, 1 completely disagree, 7 completely agree)

	1	2	3	4	5	6	7
Happy	○	○	○	○	○	○	○
Kind	○	○	○	○	○	○	○
Embarrassed	○	○	○	○	○	○	○
Troubled	○	○	○	○	○	○	○
Disappointed	○	○	○	○	○	○	○
Excited	○	○	○	○	○	○	○
Angry	○	○	○	○	○	○	○
Happy	○	○	○	○	○	○	○
Afraid	○	○	○	○	○	○	○
Satisfied	○	○	○	○	○	○	○

Regretful	○	○	○	○	○	○	○
Glad	○	○	○	○	○	○	○
Surprised	○	○	○	○	○	○	○
Sad	○	○	○	○	○	○	○
Nervous	○	○	○	○	○	○	○
Loving	○	○	○	○	○	○	○
Warm	○	○	○	○	○	○	○
Lost	○	○	○	○	○	○	○
Grateful	○	○	○	○	○	○	○
Hopeful	○	○	○	○	○	○	○

2. Do you believe the product in the advertisement has the following attributes:

	1	2	3	4	5	6	7
Delicate taste	○	○	○	○	○	○	○
Smooth and mellow	○	○	○	○	○	○	○
Delicious and sweet	○	○	○	○	○	○	○
Traditional craftsmanship	○	○	○	○	○	○	○
Long history	○	○	○	○	○	○	○
Nutritious and healthy	○	○	○	○	○	○	○

3. Do you think the following attributes of the product are important:

	1	2	3	4	5	6	7
Delicate taste	○	○	○	○	○	○	○
Smooth and mellow	○	○	○	○	○	○	○

Delicious and sweet	○	○	○	○	○	○	○
Traditional craftsmanship	○	○	○	○	○	○	○
Long history	○	○	○	○	○	○	○
Nutritious and healthy	○	○	○	○	○	○	○

4. Do you think the product in the advertisement is:

	1	2	3	4	5	6	7
Effective	○	○	○	○	○	○	○
Beneficial	○	○	○	○	○	○	○
Functional	○	○	○	○	○	○	○
Necessary	○	○	○	○	○	○	○
Practical	○	○	○	○	○	○	○
Interesting	○	○	○	○	○	○	○
Pleasant	○	○	○	○	○	○	○
Exciting	○	○	○	○	○	○	○
Enjoyable	○	○	○	○	○	○	○
Good	○	○	○	○	○	○	○
Satisfactory	○	○	○	○	○	○	○
Attractive	○	○	○	○	○	○	○
Likeable	○	○	○	○	○	○	○

5. Do you think the advertisement is:

	1	2	3	4	5	6	7
Good	○	○	○	○	○	○	○
Likeable	○	○	○	○	○	○	○

Positive	○	○	○	○	○	○	○
Pleasant	○	○	○	○	○	○	○

6. Please rate the thoughts that came to your mind while watching the ad. Please indicate your level of agreement with the following descriptions:

	1	2	3	4	5	6	7
I can relate myself to the ad	○	○	○	○	○	○	○
I think the ad is related to my own experiences	○	○	○	○	○	○	○
I am thinking about the sweet wine drinks I had before	○	○	○	○	○	○	○
I am thinking about the brewing process of sweet wine	○	○	○	○	○	○	○
The ad reminds me of my own drinking experiences	○	○	○	○	○	○	○

7. If the product is available on the market, are you likely to buy it?

Definitely	1	2	3	4	5	6	7	Definitely not
	○	○	○	○	○	○	○	
	○	○	○	○	○	○	○	
	○	○	○	○	○	○	○	
	○	○	○	○	○	○	○	
	○	○	○	○	○	○	○	
	○	○	○	○	○	○	○	
	○	○	○	○	○	○	○	

Personal Information (This information is for data statistics only and will be kept confidential)

Your gender:

○ Male

○ Female

Your age group:

○ Under 18

○ 18-25

○ 26-30

○ 31-40

○ 41-50

○ 51-60

○ Over 60

Formal Experiment Measurement Questionnaire 4

Scenario Description:

Imagine you are buying a bottle of high-quality Changle Sweet Wine for yourself. This sweet wine is an intangible cultural heritage, representing traditional brewing techniques and a unique cultural background. You highly value this purchase and hope to choose the sweet wine that best suits your taste and needs. You will carefully read product information, understand the brewing process, taste different brands, and ultimately make a well-considered purchase decision.

Instructions:

In the following tasks, please carefully consider your evaluation and choice of Changle Sweet Wine based on the provided information. Imagine you are buying this sweet wine for yourself, and your choice will directly affect your satisfaction.

Today, you will come across an advertisement for sweet wine again, and you need to obtain relevant information from the ad. Please watch the video advertisement below.

<LINK>
https://haokan.baidu.com/v?vid=4481420460012793311&pd=pcshare&hkRelaunch=p1%3Dpc%26p2%3Dvideoland%26p3%3Dshare_input

1. What emotions did you feel while watching the above advertisement? (Please indicate your level of agreement with the following emotions, 1 completely disagree, 7 completely agree)

	1	2	3	4	5	6	7
Happy	○	○	○	○	○	○	○
Kind	○	○	○	○	○	○	○
Embarrassed	○	○	○	○	○	○	○
Troubled	○	○	○	○	○	○	○
Disappointed	○	○	○	○	○	○	○
Excited	○	○	○	○	○	○	○
Angry	○	○	○	○	○	○	○
Happy	○	○	○	○	○	○	○
Afraid	○	○	○	○	○	○	○
Satisfied	○	○	○	○	○	○	○

Regretful	○	○	○	○	○	○	○
Glad	○	○	○	○	○	○	○
Surprised	○	○	○	○	○	○	○
Sad	○	○	○	○	○	○	○
Nervous	○	○	○	○	○	○	○
Loving	○	○	○	○	○	○	○
Warm	○	○	○	○	○	○	○
Lost	○	○	○	○	○	○	○
Grateful	○	○	○	○	○	○	○
Hopeful	○	○	○	○	○	○	○

2. Do you believe the product in the advertisement has the following attributes:

	1	2	3	4	5	6	7
Delicate taste	○	○	○	○	○	○	○
Smooth and mellow	○	○	○	○	○	○	○
Delicious and sweet	○	○	○	○	○	○	○
Traditional craftsmanship	○	○	○	○	○	○	○
Long history	○	○	○	○	○	○	○
Nutritious and healthy	○	○	○	○	○	○	○

3. Do you think the following attributes of the product are important:

	1	2	3	4	5	6	7
Delicate taste	○	○	○	○	○	○	○
Smooth and mellow	○	○	○	○	○	○	○

Delicious and sweet	○	○	○	○	○	○	○
Traditional craftsmanship	○	○	○	○	○	○	○
Long history	○	○	○	○	○	○	○
Nutritious and healthy	○	○	○	○	○	○	○

4. Do you think the product in the advertisement is:

	1	2	3	4	5	6	7
Effective	○	○	○	○	○	○	○
Beneficial	○	○	○	○	○	○	○
Functional	○	○	○	○	○	○	○
Necessary	○	○	○	○	○	○	○
Practical	○	○	○	○	○	○	○
Interesting	○	○	○	○	○	○	○
Pleasant	○	○	○	○	○	○	○
Exciting	○	○	○	○	○	○	○
Enjoyable	○	○	○	○	○	○	○
Good	○	○	○	○	○	○	○
Satisfactory	○	○	○	○	○	○	○
Attractive	○	○	○	○	○	○	○
Likeable	○	○	○	○	○	○	○

5. Do you think the advertisement is:

	1	2	3	4	5	6	7
Good	○	○	○	○	○	○	○
Likeable	○	○	○	○	○	○	○

Positive	○	○	○	○	○	○	○
Pleasant	○	○	○	○	○	○	○

6. Please rate the thoughts that came to your mind while watching the ad. Please indicate your level of agreement with the following descriptions:

	1	2	3	4	5	6	7
I can relate myself to the ad	○	○	○	○	○	○	○
I think the ad is related to my own experiences	○	○	○	○	○	○	○
I am thinking about the sweet wine drinks I had before	○	○	○	○	○	○	○
I am thinking about the brewing process of sweet wine	○	○	○	○	○	○	○
The ad reminds me of my own drinking experiences	○	○	○	○	○	○	○

7. If the product is available on the market, are you likely to buy it?

Definitely	1	2	3	4	5	6	7	Definitely not
	○	○	○	○	○	○	○	
	○	○	○	○	○	○	○	
	○	○	○	○	○	○	○	
	○	○	○	○	○	○	○	
	○	○	○	○	○	○	○	
	○	○	○	○	○	○	○	
	○	○	○	○	○	○	○	

Personal Information (This information is for data statistics only and will be kept confidential)

Your gender:

○ Male

○ Female

Your age group:

○ Under 18

○ 18-25

○ 26-30

○ 31-40

○ 41-50

○ 51-60

○ Over 60
