# Supplementary material for: Study on the Psychological Effects of Intangible Cultural Heritage Advertising with Different Degrees of Situational Involvement
Source: Behav Sci (Basel). 2024 Jul 22;14(7):623. doi: 10.3390/bs14070623 (PMC11274228; doi:10.3390/bs14070623)
Supplement: Supplementary file 1 [file behavsci-14-00623-s001.zip › Attachment 3 Retest Questionnaire.pdf]

Attachment Three: Retest Questionnaire

1. Please reconfirm the sweet wine product you saw in the experiment a week ago:

The brand name of the sweet wine is: [Single Choice] *

- ○ A. Yiwan Xuji
- ○ B. Jideli
- ○ C. Yiwan Chenji
- ○ D. Huatiangangzi
- ○ E. Wowo
- ○ F. Mipopo
- ○ G. Rishiji
